# A software framework for microarray and gene expression object model (MAGE-OM) array design annotation

**DOI:** 10.1186/1471-2164-9-133

**Published:** 2008-03-20

**Authors:** Matloob Qureshi, Alasdair Ivens

**Affiliations:** 1 Wellcome Trust Sanger Institute, Hinxton, Cambridgeshire, CB10 1SA, UK

## Abstract

**Background:**

The MIAME and MAGE-OM standards defined by the MGED society provide a specification and implementation of a software infrastructure to facilitate the submission and sharing of data from microarray studies via public repositories. However, although the MAGE object model is flexible enough to support different annotation strategies, the annotation of array descriptions can be complex.

**Results:**

We have developed a graphical Java-based application (Adamant) to assist with submission of Microarray designs to public repositories. Output of the application is fully compliant with the standards prescribed by the various public data repositories.

**Conclusion:**

Adamant will allow researchers to annotate and submit their own array designs to public repositories without requiring programming expertise, knowledge of the MAGE-OM or XML. The application has been used to submit a number of ArrayDesigns to the Array Express database.

## Background

Genome-wide measurement of gene expression and genomic variation via DNA microarrays is a widely used technique in biological and medical research. The quantity and complexity of data generated by such studies represents a challenge in terms of storage, analysis and data transfer. There are a number of different microarray technologies and platforms which generate data in a range of different formats and units. The Microarray Gene Expression Data (MGED) society has proposed a standard known as the Minimum Information About a Microarray Experiment (MIAME) standard to address these issues [1]. A MIAME-compliant data-centric model has been developed: the Microarray Gene Expression Object Model (MAGE-OM); this applies a structure to all components of a microarray-based study, from the sequences attached to the array through to describing equipment and platform used to perform the hybridisation reactions [2]. The MGED Ontology (MO) provides a common terminology and hierarchy for data annotation within the MAGE-OM [3]. A notable aspect of the MO is that it allows terms from existing external ontologies to be referenced within the MO framework. Additionally, an XML data-interchange format, MAGE mark-up language, MAGE-ML, has been derived from the MAGE-OM. The development of MAGE-ML facilitates the organisation and transfer of microarray data between databases and a range of analysis programs [2].

There are three public repositories of data produced from microarray studies which are recommended by the MGED society: ArrayExpress [4], CIBEX [5] and Gene Expression Omnibus (GEO) [6]. These databases have various differing data submission interfaces. Data can be supplied to ArrayExpress via ADF files, MIAMExpress [7] web-forms or via MAGE-ML generated with Tab2Mage [8]. GEO can accept submissions via a web interface, a tab-delimited format, Simple Omnibus Format in Text (SOFT) and an XML format based around SOFT, MiniML [9]. CIBEX accept submissions via a Microsoft Word document form and also provides a Java tool compatible with MS-Windows and Mac OSX [10]. Submissions to ArrayExpress and GEO can currently be made in MAGE-ML format. An increasing number of array data analysis software packages are also capable of importing and exporting MAGE-ML files. For example, the RMAGEML Bioconductor package for the R statistical computing environment allows the importation of experiment design in the form of MAGE-ML annotation [11].

MAGE-ML is a flexible and extensible annotation format, allowing information to be structured in a variety of ways. This flexibility can influence the complexity of software and databases designed to read and store such data. Therefore, ArrayExpress and GEO give guidance on the coding of MAGE-ML in order to facilitate submission in this format [12].

Here, we describe an application for the annotation of descriptions of microarray designs in a MIAME-compliant manner in the form of MAGE-ML or other file formats used by the public repositories: Array Design MAGE-ML Annotation Tool (Adamant). The application interfaces do not rely on user knowledge of the MAGE-OM but are flexible enough to allow annotation of complex multi-species microarrays. It also makes full use of the MGED Ontology, with ontology terms and documentation available at all appropriate steps of the annotation process. The application is capable of exporting data in SOFT, MAGE-ML and MAGE-TAB format [13].

## Implementation

### Requirements

We set out to design a simple interface for annotation of the ArrayDesign and associated packages from the MAGE-OM that would produce data files for submission to ArrayExpress or other public repositories.

The Adamant software should be capable of execution on a variety of platforms without needing to install databases and/or other software. As data annotation can sometimes be a lengthy process, a facility to save work in an incomplete state prior to completion should also be incorporated.

Software implementations of the MAGE-OM are available in Perl and Java and are referred to as MAGE software toolkits (MAGE-stk) [14]. These toolkits are capable of producing MAGE-ML representing the various MAGE-OM packages.

### Implementation

The Java language was chosen for development of Adamant, primarily due to the availability of user interface components in the Swing library, the ease of data caching, and the existence of the Java MAGE-stk for MAGE-ML output. Existing open source Java packages are used to assist processing wherever possible.

**Figure 1:**
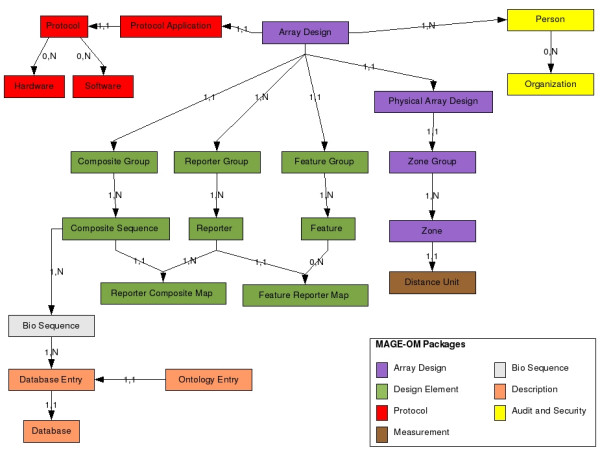
Diagram showing the classes from the MAGE-OM implemented in the application. The lines show key relationships between the various classes and the numbers show cardinality constraints whilst the arrows indicate the directionality of the constraint. The colour of the class box indicates which MAGE-OM package the classes are from. The Connections to the Ontology Entry box are not shown to preserve clarity. The Ontology Entry class is actually referenced by many of the classes shown in the diagram.

The MAGE-OM packages necessary for the annotation of the DNA Sequences attached to a microarray are the ArrayDesign, BioSequence, DesignElement, Database, Protocol and Description packages. The majority of annotation information is organised into the former three packages, whilst the Database and Description packages describe relationships to public databases and terms from the MO (Figure 1) and other ontologies, e.g. life cycle ontologies appropriate to a given organism.

The MAGE-OM models relationships between various classes in a flexible but complex way. Because representation of classes in this form makes update and changes to objects a multi-step process, a series of proxy classes, which map to MAGE-OM classes, was implemented. The relationships between objects are stored in a set of classes created to enable bi-directional connections between objects to be stored and retrieved efficiently. Software design patterns such as Model view controller (MVC) and Flyweight are used where appropriate throughout the software [15]. GUI components are designed to be reusable in a variety of contexts. For example, the ontology browser component allows the appropriate branch of MO to be viewed and instances to be selected or new instances created in a number of different contexts during the annotation process. Adamant is also structured to allow sharing of MAGE-OM components such as ontology entries and protocols between ArrayDesign annotations. As a result, short MAGE-ML fragments, which would normally be considered invalid without the context of the appropriate MAGE-OM package, can be stored and transferred between different ArrayDesign annotations.

The MAGE-OM allows certain data to be represented in more than one way, which thus affects the complexity of processing MAGE-ML files. The MAGE-ML output of the application is designed to be compatible with best-practice for submission to ArrayExpress [12]. We have also followed proposals with relation to usage of identifiers with a Life Sciences Identifier (LSlD)-like structure so that objects referenced in the MAGE-ML files have unique identifiers [16]. As the MAGE-OM provides a complex and complete framework for representation of data in a MIAME-compliant manner, the application is also able to produce output files in the SOFT format created by GEO or the proposed MAGE-TAB format.

## Results

### Initialisation and configuration

Adamant is designed to be distributed as a jar file zipped together with the Java Mage toolkit, the Jena ontology handling framework [17] and the Ehcache object caching framework [18] as jar files.

On invocation within the Java Virtual Machine (JVM), the application first loads various configuration settings. Default values for the configuration settings are provided and a GUI form allows editing of these values. The configuration settings are stored in a file and this also can be edited in a text editor. The configuration file contains an entry pointing to the location of the MGED Ontology file in OWL format.[19] This can be a URL to the MGED Ontology source.forge www site or to a local copy of the ontology. The default behaviour is to retrieve the Ontology via the internet, ensuring that ontology entries presented during annotation are current. The ontology file is read using the open source Jena ontology package. As processing of the Ontology can slow down the startup time, one of the configuration settings allows the ontology to be stored in a persistent cache, speeding up the start time of successive instances of the software. Other configuration settings include proxy server settings and an entry for the identifier prefix in order to conform to the ID guidelines proposed by MGED.

### Loading array description files

The starting point for annotation of an Arraydesign is defining the properties of the material spotted or printed on the microarray slide. These are termed Features in MAGE-OM and can be grouped into one or more zones within an ArrayDesign. The features are then mapped to reporters, which represent properties of the actual sequence spotted onto the array. The reporters are in turn mapped to BioSequences which provide a mechanism for relating reporter sequences to genes or other features of interest whilst also allowing mapping to data in public sequence repositories (Figure 1).

Features and reporters are created by loading an array design file such as the gal file format used by GenePix [20]. This file contains two sections: the header defines the number and location of all the subgrids on the array, whilst the second section defines the locations of all the spots within the subgrids. The ID column in the latter section is used to create reporters which are linked to features at a given location. Spots containing identical sequence can be represented by allocating the same identifier within the second tab-delimited table. The Java framework for reading array definitions uses interfaces to ensure that file readers for other array formats can be added as required.

We have used the Ehcache object caching framework [18] to ensure the application is scaleable from small to very large ArrayDesigns.

### Creating ontology instances

**Figure 2:**
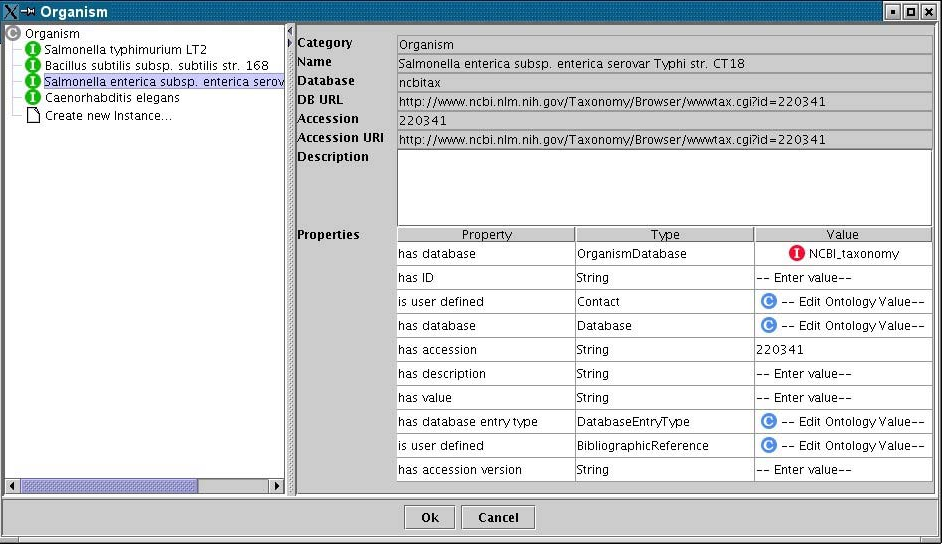
The ontology selection and editing component. This component is made up of two panes. The left pane allows any branch of the MGED Ontology to be viewed as a tree. The relevant sub-branch of the ontology is displayed during annotation. Classes containing subclasses or instances can be clicked to open tree views of the contents. The right panel gives details on any class or instance that is selected. This includes a description taken from the MGED Ontology and a list of properties.

The MGED ontology is retrieved via the internet or local storage, and resolved in memory during initialisation. When an ontology entry is required the appropriate section of the ontology is displayed in a pane-window GUI component. Although the ontology is actually a directed acyclic graph, it is represented as tree nodes connected to multiple parents, shown as leaves at each relevant branch of the tree. Any nodes with multiple parents are represented as redundant branches from the main tree. The GUI component for editing and browsing the MGED-Ontology is re-used throughout the annotation process (Figure 2). The left pane shows a tree view of the Ontology branch, allowing opening of subclasses and the selection of predefined instances. The colour of the instance icon indicates whether it is part of the MGED Ontology or a user-defined addition. The right pane shows properties of the ontology including a description and a list of restrictions. If an instance is selected in the left pane, the restrictions on the ontology are editable. Ontology selections and user-defined instances created with this GUI-component can be exported as MAGE-ML OntologyEntry fragments. Whilst these fragments are not valid MAGE-ML, they provide a convenient mechanism to allow sharing and re-use of ontology terms between users of the annotation interface.

### Annotation of Reporters and BioSequences

**Figure 3:**
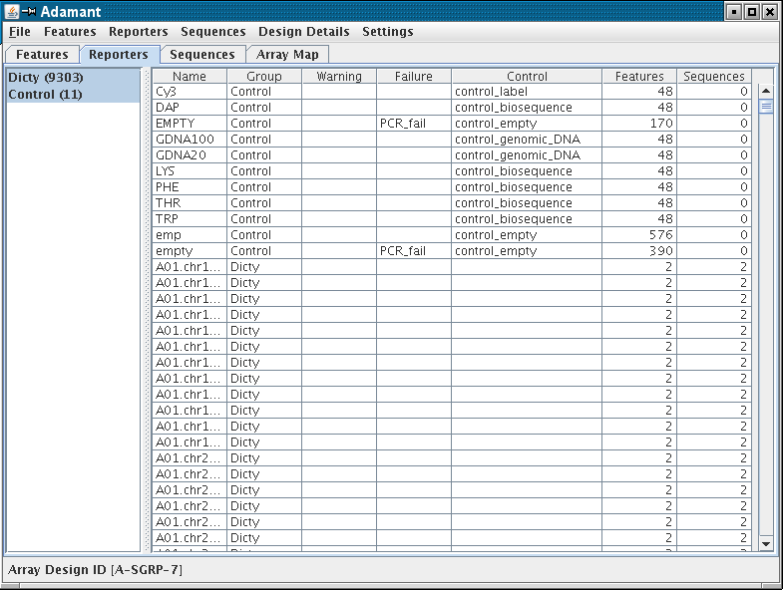
The Reporter view. This two panel component allows creation and editing of ReporterGroups and the assignment of Reporters to ReporterGroups as well as editing of the various properties of Reporters. The left panel lists ReporterGroups and the right panel shows a table of the Reporters in the groups

The user interface displays a tabbed series of windows to allow viewing of Features, Reporters and BioSequences. All Reporters and Features must be members of a ReporterGroup or FeatureGroup, respectively. The Reporter and Feature windows use a 2 pane display: the left-hand pane shows the groups and allows creation, editing and deletion of the appropriate type of group (Figure 3). The "create and edit" dialog windows show fields appropriate for the type of group being created. The right-hand pane shows the individual Features or Reporters as rows in a table. Any column can be double-clicked to sort the rows according to values in that column. Ascending and descending ordering can be alternated by successive double-clicks on a column. Groups of Reporters can be selected and edited to change the properties.

**Figure 4:**
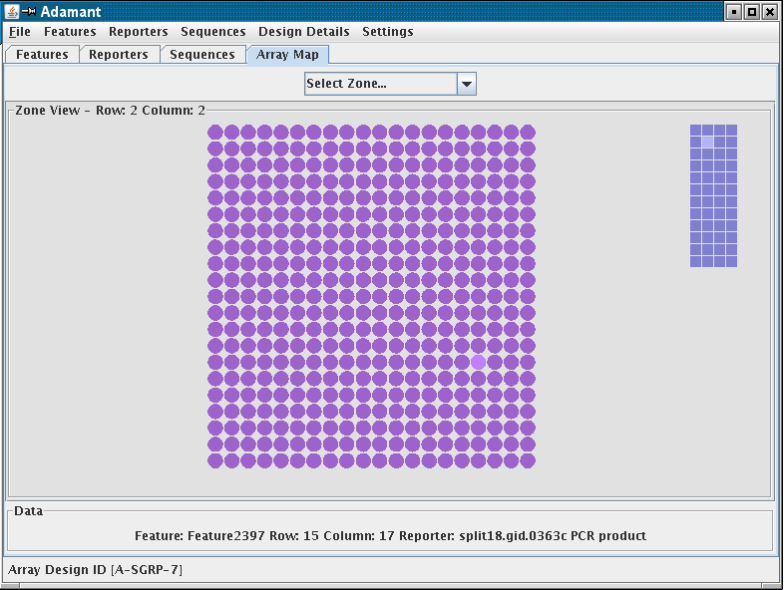
The Zone view. This component can display the array as a series of Zones or the Features within a zone. The figure shows the view within a Zone. Moving the mouse pointer over a circle representing a Feature results in information about the feature being shown in the Data section. Double-clicking a circle opens a Feature-Reporter editor component. The array of squares to the right of the features allows easy navigation to other zones in the array.

The elements of the microarray can also be viewed in the ArrayMap tab which shows a diagrammatical view of the Array (Figure 4). The first view shows the subgrids, known as Zones in the MAGE-OM. Any Zone can be clicked to give a view of the Features or spots within that subgrid. The lower information panel shows details of any spot the mouse pointer is moved to. Clicking on a spot in the Zone view allows the Reporter associated with that particular Feature to be edited. A smaller schematic of the array as a series of rectangles in the zone view allows easy navigation to other zones.

Where duplicate Reporters have been given unique names within the array definition file, or there is a need to map a collection of features to a single reporter, a custom tab-delimited file containing the identifiers to be mapped can be loaded to allow Feature-Reporter mapping to be merged. Alternatively individual Feature rows can be selected and mapped to a Reporter identifier.

BioSequence data can be mapped to Reporters by creating individual BioSequences in the Reporter tab window or via a tab-delimited file. Once created, BioSequences can be selected and edited in the same manner as Reporters.

### Annotation of protocols and associated information

The MAGE-OM provides a number of classes for the description of protocols. These include classes for Hardware and Software used in a protocol. The annotation interface includes several GUI components for creating and adding Array Manufacture protocols, which are required for submission of an ArrayDesign. The protocols can also be exported to files, shared or imported as MAGE-ML fragments in the same manner as OntologyEntry files. GUI forms are also provided to allow entry of DesignProviders and various details about the ArrayDesign required for MIAME-compliance.

### Producing MAGE-ML files

The output of MAGE-ML is handled by classes from the Java MAGE-stk. The internal representations of data are mapped to MAGE-OM objects, with appropriate relationships being created. Some validation is also performed at this stage to ensure that the MAGE-ML produced will be complete. The Adamant software warns the user of any missing information, providing guidance about what needs to be added. [Adamant also provides the ability to run and view the output of the ArrayExpress MAGE-ML validation tool [21] to check the data will be acceptable to ArrayExpress. Any errors are output to a dialog window and a log file.]

### Output in SOFT and MAGE-TAB format

Output of data in GEO SOFT or MAGE-TAB formats is in accordance with the relevant specifications [9,12], using modules within Adamant.

## Discussion

### Comparison

The flexibility and complexity of the MAGE-OM and associated databases has made the task of coding transferring data from data providers to data storage a difficult one. There are a number of methods for submitting Array Designs to repositories such as ArrayExpress. Interfaces involving interactions with web forms are time consuming, complex and error prone. Other methods, such as production of tab-delimited tables, are not sufficiently flexible to allow annotation of certain types of ArrayDesign, or require detailed knowledge of the MAGE object model. The annotation interface we have developed offers a relatively simple set of interfaces, with the rich feature-set provided by the Java Swing libraries; it is flexible enough to annotate complex multi-species Array Designs. We have kept dependencies to a minimum, thus users can install and run the software without needing to install relational databases or other additional packages.

We have used the Adamant software to submit four array designs to Array Express in MAGE-ML format (A-SGRP-1 to A-SGRP-5). The submission process, undertaken with guidance from the Array Express curation team, has helped to refine the development of the interfaces and validation of MAGE-ML output.

## Conclusion

Adamant will run on any platform supporting the Sun Java 1.4 Virtual Machine. The CPU and memory requirements will vary, depending upon the size and complexity of the ArrayDesigns being annotated, but the use of EhCache ensures that large ArrayDesigns can be edited on a typical Desktop machine with 1 Gb of RAM. The software makes use of the Jena and MAGE-stk libraries which are released under the GNU-licence and the software itself is released under the same open-source licence.

The interface components have been structured to collect all the data necessary to conform to MIAME guidelines; a variety of compliant, text-based file output formats can be generated. Adamant could also directly interface with a suitably structured database via the Java Data Base Connectivity (JDBC) library.

The annotation software presented here has been designed as a set of reusable Java Swing components. A subset of the components readily lend themselves to the annotation of other MAGE-OM packages such as Experiment and BioMaterial which are required for the submission of experimental data to public repositories.

## Availability and requirements

Project name: Microarraykit

Project home page: 

Operating system(s): Any platform with a Java 1.4 compatible JVM.

Programming language: Java

Other requirements: Java 1.4

License: GPL

Executable code is available in additional file 1, and test data in additional file 2.

## Authors' contributions

MQ carried out the design and implementation of the software and wrote the manuscript. AI participated in setting requirements, testing the software and suggesting improvements. Both authors read and approved the final manuscript.

## Supplementary Material

Additional file 1 Adamant executable code. This file contains the java files and scripts needed to run the Adamant software. Click here for file

Additional file 2 Test data. This file contains test data and a walkthrough of an annotation with Adamant. Click here for file
