# Supplementary material for: A software framework for microarray and gene expression object model (MAGE-OM) array design annotation
Source: BMC Genomics. 2008 Mar 20;9:133. doi: 10.1186/1471-2164-9-133 (PMC2358904; doi:10.1186/1471-2164-9-133)
Supplement: Additional file 1 — Adamant executable code. This file contains the java files and scripts needed to run the Adamant software. [file 1471-2164-9-133-S1.gz › adamant.pdf]

Adamant (Array Design MAGE-ML Annotation Tool) manual

Introduction
Adamant is a Java application to enable MIAME compliant annotation of Array Designs. The application produces output files in MAGE-ML, MAGE-Tab and SOFT formats which are accepted by public repositories of microarray data.

Configuration
The behaviour of Adamant can be configured to make use of a network connection to download the latest version of the MGED-Ontology. Configuration options can be edited from the settings menu. Options include the ability to set a web proxy and caching of the ontology between application runs.

MO ontology editor
The application makes use of the MO ontology throughout the annotation process. Where an ontology term is required, the ontology button shows the class name from which an instance should be selected. On clicking the button a split pane view of the ontology class and any existing instances is displayed. The final option in the tree view allows creation of a new instance.

Selecting an instance or subclass results in the right pane displaying information on the selected item. Selecting the “create new instance” option gives allows a new ontology instance to be defined. This is how references to other external ontologies can be made from within the MGED Ontology.

Ontology terms defined in this way can be exported and imported as MAGE-ML ontology fragments via the “File” menu.

Load Gal File
An array design description file such as a GAL file is required for creation of sub grids (zones) and identification of spot data (features) within the zones. The “Load Gal File” option is selected from the File menu. This brings up a file chooser dialog window to select a gal file.

The “Gene Name Column” box on the right of the directory view allows selection of the Column heading which contains identifier data. In old gal files this was stored in the ID column but most gal files store this information in the Name column.

Features and Feature Groups
The Features tab displays features in table, where each row represents spots on the array. The location of the spot within a zone and position of the zone within the array are shown here.

All features are grouped into a single feature group. More feature groups should be created to separate features if different technology types have been printed

on the array e.g. a mixed array of oligonucleotide probes and PCR sequences. The “ReporterName” column shows the identifier associated with the feature data.

Reporters and Reporter Groups

The Reporters tab shows a tabulated view of Reporter where each row represents a the contents of each spot on the array. If the array contains multiple Features containing the same material, the reporter view will show one row corresponding to all such features. The Features column in the Reporters table shows the number of Features which have mapped to the Reporter.

Reporters can be edited to associate MO ontology entries with the Reporter. These are used to indicate warnings, failures or control types. Reporters can be combined where they represent the same material spotted on the array by selecting both rows and right clicking and selecting the “merge” option.

Reporters are all assigned to a single ReporterGroup but more groups should be created to distinguish control and experimental spots. Further groups may be necessary to separate species specific probes. Ontology terms for the Organism field can selected and edited via the Reporter Group edit dialog window.

Reporter data can be exported as a text file. The field delimiter can be selected from the right hand “CSV separator” box. Data may be edited in another application and imported back in. Reporters may also be combined by importing a reporter merge file.

Array Map

The array map tab gives a schematic view of the arrangement of zones and spots on the array. The “array view” allows a zone to be selected and window switches to show the “zone view” where circles corresponding to features can highlighted. A smaller version of the array view is displayed to the right of the zone (where window size permits it).

Double clicking on a spot brings up an edit dialog box where the reporter data associated with the feature can be edited. Changes made here affect all features which map to the same reporter.

Sequence Files

Sequence data is associated with Reporters by loading a sequence file. This is a text file with a number of columns identifying which Reporter name the sequence maps to, as well as columns describing properties of the sequence itself.

The columns are

- Reporter
  - This column is mandatory and contains a pre-existing Reporter Name.
- Sequences

    - This is also mandatory and defines a unique name for the new sequence.
- Sequence-type
    - This column will contain ontology terms defining the the nature of the sequence. These will normally correspond to entries in the SO (sequence ontology).
- Sequence-Polymer
    - The sequence polymer column contains ontology terms describing the nature of the molecule being described e.g. DNA or RNA.
- Sequence-Annotation
    - The annotation column can contain the values “Attached” to indicate a sequence which represents material spotted directly on the array or “Database” where the sequence refers to a sequence element stored in a public sequence database but not the actual sequence spotted on the array.
- Sequence-Organism
    - This column will contain ontology terms defining the species from which the sequence is derived.
- Sequence-Description
    - This column can contain optional description information.
- Sequence-Sequence
    - The actual sequence string is stored in this column.
- Database
    - The name of the database containing further sequence data.
- Database-Version
    - An optional column describing the database version
- Database-URI
    - The URI of the database being described.
- Database-Description
    - An optional description of the database.
- Database-Entry
    - The accession number or identifier associated with the sequence.
- Database-Entry-URI
    - The URI of data corresponding to the sequence.

Sequences

The sequence table displays the sequences imported from the sequence file. Sequences can be selected and edited via this table. Sequence data may be exported and further edited before being imported.

Array Design Details

The “Design details” menu contains the “Array Design Details” option. This brings up the Array Design Details dialog box. Various aspects of the array design such as Identifier, Name, Version and Description can be edited here. The “surface type” button allows an ontology term to to be selected or created and associated with the ArrayDesign.

Protocols

Protocols are added to the array design via the “Design details” menu. The dialog box allows editing or creation of new protocols. Software and Hardware data can also be linked to a protocol via the Software and Hardware list boxes.

Protocols can be exported or imported as XML files via the “File” menu.

Contacts

Contacts represent people and organisations involved in the production of the array design or annotation file. These can be created and edited via the Contacts option in the “Design details” menu.

Contacts can also be exported or imported as XML files via the “File” menu.

Saving/loading data

Annotation can be saved and restarted at any time by selecting the “save array design” and “load array design” options of the “File” menu respectively. The array design data saved via these options is in a binary format used only by the Adamant application.

MAGE-ML

MAGE-ML XML output can be generated from a complete annotation via the “Write MAGE-ML file” option of the “File” menu. This option makes heavy use of the MAGE-stk which may require a large amount of free memory, depending on the size of the array and type of annotation. More memory can be allocated to the application by invoking the Java virtual machine with a larger -Xmx e.g. option. This can be edited in the adamant.sh (for unix variants) or adamant.cmd (MS windows) file.

Validation

A MAGE-ML file can be validated by the MAGEloader software from the Arrayexpress group. Adamant contains the option to run the validator on a selected MAGE-ML file. Once a file has been selected the run.sh file of the MAGEloader must be located. The MAGEloader is then called from within Adamant and the resulting files can be viewed in the log viewer dialog window.

The MAGEloader must be configured by invoking run.sh prior to running it from the Adamant application.

MAGE-Tab and SOFT

MAGE-tab and SOFT tabular output files can be produced by choosing the appropriate option from the “File” menu.

Walk through with some sample data

The data for this walk through can be found in the testdata subdirectory

1. Start the application by invoking adamant.sh (Unix/Linux/MacOS) or adamant.cmd (MS Windows)
2. Select File and Load Gal file from the menu. Select the file **dicty.gal** from the testdata subdirectory.
3. Select File and import ontologies to use the predefined Sequence Ontology and Organism terms during annotation. These are in the files **dicty-onotology.xml** and **so-terms.xml** in the testdata subdirectory.
4. Click on the Reporters tab and create a new Reporter Group by right clicking in the Reporter Group panel on the left
5. Name this group as Control. Click the Group Type text and select **control_design_element_group_type** from the ontology window display.
6. Double click on the "Features" column in the Reporter Table view and scroll down until reporters mapping to more than four features are displayed. Select these reporters and use the "send to" sub menu to move them to the new Controls ReporterGroup.
7. Select the reporter rows, right click and select the edit selected reporter properties option. Select the appropriate ontology terms for the reporters.
8. Edit Reporter Group1 so that it's type is **experimental_design_element_group_type** and the Organism is **Dictyostelium discodeum**.
9. Attach sequence data to the reporters by selecting load sequence file from the Sequences menu. Select the file **dictyseqs.txt** from the testdata directory.
10. Import a protocol by selecting the appropriate option from the File menu and selecting the file, **dicty.protocol** in the testdata directory. The protocol can be viewed and edited from the Protocols option in the design details menu.
11. Import contacts in a similar way by loading the file, **dicty.contact**. Contacts are edited from the Contacts option in the Design Details menu.
12. The array design form is displayed via the Design details menu and this is where the identifier for the Array design and other data are entered.
13. Save Array Design Files via the File menu.
14. Export MAGE-ML by selecting the action from the File menu. As this is a computationally intensive process, production of the file can take a few minutes depending on the available CPU and memory. The output of this walk through is saved as mage in the file **dictytest.xml**.
15. This file can be validated using the MAGEloader available from the European Bioinformatics institute website (ftp://ftp.ebi.ac.uk/pub/databases/microarray/code/loader-validator/). The validator can be run as a standalone application or it can be called from within Adamant by selecting the option to "Validate MAGE-ML file" from the File menu. As the MAGEloader requests configuration information the first time it is run, we recommend running it in standalone mode before calling it

from within Adamant. The validation logs will show if the validation was successful.

16. If this walk through is followed with your own data. The validated MAGE-ML file will now ready for submission to a public repository. Please check the submission information at the repository of your choice.

Data submission guidelines for Public Repositories

ArrayExpress http://www.ebi.ac.uk/microarray/submissions.html
GEO http://www.ncbi.nlm.nih.gov/projects/geo/info/submission.html
CIBEX https://cibex.nig.ac.jp/submission/overview.jsp
