# Supplementary figures and images for: A software framework for microarray and gene expression object model (MAGE-OM) array design annotation
Source: BMC Genomics. 2008 Mar 20;9:133. doi: 10.1186/1471-2164-9-133 (PMC2358904; doi:10.1186/1471-2164-9-133)

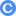

Supplement: Additional file 1 — Adamant executable code. This file contains the java files and scripts needed to run the Adamant software. [file 1471-2164-9-133-S1.gz › resource/icon_class.png]

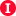

Supplement: Additional file 1 — Adamant executable code. This file contains the java files and scripts needed to run the Adamant software. [file 1471-2164-9-133-S1.gz › resource/icon_instance.png]

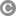

Supplement: Additional file 1 — Adamant executable code. This file contains the java files and scripts needed to run the Adamant software. [file 1471-2164-9-133-S1.gz › resource/icon_class_open.png]

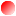

Supplement: Additional file 1 — Adamant executable code. This file contains the java files and scripts needed to run the Adamant software. [file 1471-2164-9-133-S1.gz › resource/icon_bg.png]

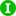

Supplement: Additional file 1 — Adamant executable code. This file contains the java files and scripts needed to run the Adamant software. [file 1471-2164-9-133-S1.gz › resource/icon_user_instance.png]
